# Effectiveness of treatments for acute and sub-acute mechanical non-specific low back pain: protocol for a systematic review and network meta-analysis

**DOI:** 10.1186/s13643-019-1116-3

**Published:** 2019-08-08

**Authors:** Silvia Gianola, Greta Castellini, Anita Andreano, Davide Corbetta, Pamela Frigerio, Valentina Pecoraro, Valentina Redaelli, Andrea Tettamanti, Andrea Turolla, Lorenzo Moja, Maria Grazia Valsecchi

**Affiliations:** 1 Unit of Clinical Epidemiology, IRCCS Istituto Ortopedico Galeazzi, Milan, Italy; 2 Department of Biomedical Sciences for Health, University of Milan, Milan, Italy; 3 Center of Biostatistics for Clinical Epidemiology, School of Medicine and Surgery, University of Milano-Bicocca, Monza, Italy; 4 Epidemiology Unit, Agency for Health Protection of Milan, Milan, Italy; 5 Rehabilitation and Functional Recovery Department, IRCCS San Raffaele Hospital, Milan, Italy; 6 Vita-Salute San Raffaele University, Milan, Italy; 7 Child and Adolescent Neuropsychiatric Unit, ASST Grande Ospedale Metropolitano Niguarda, Milan, Italy; 8 Department of Laboratory Medicine, OCSAE, Azienda USL of Modena, Modena, Italy; 9 Casa di Cura del Policlinico di Milano, Milan, Italy; 10 Laboratory for Neurorehabilitation Technologies, Fondazione Ospedale San Camillo IRCCS, Venice, Italy

## Abstract

**Background:**

Non-specific low back pain (LBP) is the leading cause of disability worldwide. Acute LBP usually has a good prognosis, with rapid improvement within the first 6 weeks. However, the majority of patients develop chronic LBP and suffer from recurrences. For clinical management, a plethora of treatments is currently available but evidence of the most effective options is lacking. The objective of this study will be to identify the most effective interventions to relieve pain and reduce disability in acute and sub-acute non-specific LBP.

**Methods/design:**

We will search electronic databases (MEDLINE, Embase, CENTRAL) from inception onwards. The eligible population will be individuals with non-specific LBP older than 18 years, both males and females, who experience pain less than 6 weeks (acute) or between 6 and 12 weeks (subacute). Eligible interventions and comparators will include all conservative rehabilitation or pharmacological treatments provided by any health professional; the only eligible study design will be a randomized controlled trial. The primary outcomes will be pain intensity and back-specific functional status. Secondary outcomes will be any adverse events. Studies published in languages other than English will also potentially be included. Two reviewers will independently screen the titles and abstracts retrieved from a literature search, as well as potentially relevant full-text articles. General characteristics, potential effect modifiers, and outcome data will be extracted from the included studies, and the risk of bias will be appraised. Conflicts at all levels of screening and abstraction will be resolved through team discussions. After describing the results of the review, if appropriate, a random effects meta-analysis and network meta-analysis will be conducted in a frequentist setting, assuming equal heterogeneity across all treatment comparisons and accounting for correlations induced by multi-arm studies using a multivariate normal model.

**Discussion:**

Our systematic review will address the uncertainties in the use of pharmacological or non-pharmacological treatments, and their relative efficacy, for acute and subacute LBP. These findings will be useful for patients, healthcare providers, and policymakers.

**Systematic review registration:**

PROSPERO CRD42018102527

**Electronic supplementary material:**

The online version of this article (10.1186/s13643-019-1116-3) contains supplementary material, which is available to authorized users.

## Background

Low back pain (LBP) is considered a symptom, and not a disease [1]. Various spinal structures including ligaments, facet joints, paravertebral musculature and fascia, intervertebral discs, and spinal nerve roots have been implicated as pain generators [2]. Nevertheless, 85% of patients with isolated back pain still do not have a definitive cause identified for their symptoms [3]. The aetiologies can be subdivided into mechanical, systemic, and referred groups. By far, the most frequent cause is mechanical (97%) [2] with the most common form of “non-specific LBP” [4]. This definition is used when the cause of the pain cannot be precisely determined [1] and is based on the exclusion of patients with a specific cause (e.g., fracture, infection, cancer) [4].

Non-specific LBP is commonly defined as pain or discomfort localized in the area of the posterior aspect of the body, from the lower margin of the twelfth rib to the lower gluteal folds, with or without pain referred into one or both lower limbs, which lasts for at least 1 day [5]. Non-specific LBP is classified by the duration as acute (pain lasting less than 6 weeks), sub-acute (6 to 12 weeks), or chronic (more than 12 weeks) [6]. Acute LBP is one of the most common reasons for adults to see a general practitioner because of moderate to severe pain and debilitating motor and psychological functions [7]. The worldwide point prevalence of LBP is 9.4% (95% CI, 9.0–9.8) in 2010 and is higher in males and the elderly, exceeding 30% in 80-year-old men in Europe [5].

Despite its widespread occurrence, acute LBP is considered to be typically self-limiting, with a recovery rate of 90% within 6 weeks of the initial episode [8], whereas 2 to 7% of patients develop chronic LBP and have a high risk of recurrence [4, 8]. The progression to chronicity is associated with high disability and costs for society [9]. Out of all 291 conditions studied in the Global Burden of Disease 2010 Study, LBP ranked highest in terms of disability and sixth in terms of overall burden expressed as disability-adjusted life-year (DALYs). Estimated DALYs increased from 58.2 million in 1990 to 83.0 million in 2010 [5]. In fact, LBP leads to a greater number of people leaving the labor force than diabetes, hypertension, neoplasm, asthma, and heart and respiratory disease combined [10].

There are many different therapeutic interventions for acute and sub-acute non-specific LBP, including pharmacological and physiotherapy treatments that are sustained by several systematic reviews [11–22]. However, none of them has been universally accepted as being the most efficacious. The five most recent guidelines (from 2015 to 2018) developed inconsistent and discordant recommendations for acute LBP [23, 24]. The uncertainties regarding the most effective treatment may be due to the absence of multiple direct comparisons of the available treatments. In fact, the majority of published studies compare only two interventions at a time. It would be helpful for clinicians, patients, and all stakeholders to know the relative efficacy of all the available treatments for acute LBP in terms of benefits and harm, in order to inform treatment decision and let to choose the best option on the basis of evidence and not only according to expert opinion.

We therefore plan to carry out a comprehensive systematic review of the acute non-specific LBP interventions to evaluate, through a multiple-treatment meta-analysis, the contribution of the current therapeutic options used to treat these patients and offer a rank of being the best among the available treatments.

The objective of this systematic review will be to assess the effectiveness of treatments for acute and sub-acute mechanical in adults with LBP.

## Methods

A systematic review protocol has been developed and registered with the PROSPERO database (CRD42018102527). This review protocol was prepared using the Preferred Reporting Items for Systematic Review and Meta-Analyses Protocol (PRISMA-P) guidelines and their recommendations [25, 26]. We have completed the PRISMA-P checklist (Additional file 1). Additional sections specific to NMA have been considered according to Chaimani et al. [27]. We will use the PRISMA-NMA extension statement to structure the contents of the actual systematic review and network meta-analysis [26].

### Eligibility criteria

#### Types of studies

We will only include randomized controlled trials (RCTs) excluding quasi-randomized trials. Cross-over randomized trials will be excluded since they are inappropriate study design for acute mechanical LBP. Studies will be considered as RCT if authors explicitly state that it is randomized [28].

#### Participants

We will include trials that involve participants older than 18 years, both males and females, experiencing pain for up to 12 weeks of non-specific LBP. We will classify the population based on pain duration: acute (less than 6 weeks) or subacute (6 to 12 weeks) [4]. Accordingly, we will select trials for pain duration, regardless of the population definition reported for a study (e.g., chronic patients with pain for less than 12 weeks). When the duration of pain allowed in the primary study, as the inclusion criteria, exceeds for a few weeks the standard definition of subacute pain (i.e., recruitment from 8 to 16 weeks) and the appropriate subgroup data is not reported in the publication as a subgroup, we will contact the authors to obtain the data for our population of interest only. If the investigators will not provide the data, the study will be excluded. According to the definition of aspecific LBP, we will exclude studies focusing on specific pathological entities (e.g., spondylolisthesis) and subgroups of patients (e.g., pregnant women). There will be no restriction on the severity or stage of the symptoms. Studies focusing on both neck and back pain in which the two subgroups of patients cannot be identified, or patients presenting with both conditions, will be excluded.

#### Interventions

We will consider all conservative rehabilitation or pharmacological treatments provided by health professionals, such as general medical practitioners or physiotherapists, aimed at relieving pain and/or reducing physical disability. We will consider any modality (e.g., physical, pharmacological), treatment extent, frequency, or intensity. We will exclude RCTs or arms of RCTs including non-conservative treatments (e.g., surgical approaches), herbal medicine, homeopathy, and all alternative treatments except for acupuncture and dry needling. We will include them since they could be clinically relevant for LBP stakeholders and there is sparse evidence of their efficacy for acute LBP in the literature [22, 29–31]. We will extract sufficient and important intervention details as suggested by the TIDieR checklist [32] in order to create consistent nodes [33–35]. Thus, we will set the following classification of interventions for potential nodes:

1. Biopsychosocial rehabilitation (including cognitive behavioral treatment and back school)
2. Exercise (e.g., resistance or aerobic training)
3. Manual therapy (e.g., spinal manipulation, mobilization, trigger point/myofascial therapy)
4. Dry needling and acupuncture
5. Education (e.g., booklet)
6. Any physical therapy (e.g., low-laser therapy, diathermy, transcutaneous electrical nerve stimulation, ultrasound therapy, heat wrap)
7. Taping (e.g., kinesiotaping)
8. Usual care defined as treatment suggested by general medicine (minimal intervention: advice to stay active or to take drugs as needed)
9. Paracetamol
10. Non-steroidal anti-inflammatory drugs (NSAIDs), including COX-2 inhibitors
11. Muscle relaxant drugs
12. Opioid drugs
13. Steroids
14. Antidepressant drugs
15. Inert treatment (e.g., placebo drug, sham therapy)
16. No treatment (no treatment, waiting list control)

For the exercise, education, manual therapy, and physical therapies nodes, we will explore any kind of therapy with own delivered modality. If enough studies share the same description of the intervention (assessment made by the TIDieR checklist [32]) that allows the creation of a new node, we will build a new subgroup category as follows: (I) exercise: as example, for instance, single supervised or home exercise, stretching, aerobics, or resistant training; (II) manual therapies: as example, for instance, mobilization and manipulation, trigger points, and muscle therapy; (III) education: as example, for instance, booklet/advices, ergonomics, and workplace interventions; (IV) physical therapies: as example, for instance, low-laser therapy, diathermy, transcutaneous electrical nerve stimulation, ultrasound therapy, and heat wrap.

#### Outcomes and study time points

Primary outcomes will be pain intensity (e.g., measured by numeric rating scale, visual analog scale, McGill Pain Questionnaire or, box scale, other validated quantitative measures) and back-specific functional status (e.g., measured by the Oswestry disability questionnaire, Roland-Morris disability scale or other validated quantitative measures). If a trial reports more than one measure of pain intensity in different conditions (e.g., “night” or “at rest” or “at movement”), we will select “pain at rest” as a measure of generic pain. The secondary outcome will be any adverse event. We will define the adverse events (AE) based on the grade of severity. The Common Terminology Criteria for Adverse Events displays grades 1 through 5 with unique clinical descriptions of severity for each AE based on this general guideline: grade 1 mild AE, grade 2 moderate AE, grade 3 severe AE, grade 4 life-threatening or disabling AE, grade 5 death related to AE. We will classify the AE a posterior, since we expect differences in the nature of events according to the type of intervention (pharmacological or not pharmacological) [36].

All time points will be abstracted. However, in the analyses, we plan to summarize the immediate-term (closest to 1 week), short-term (closest to 1 month assessment), intermediate (closest to 3–6 months), and long-term effects (closest to 12 months).

### Information sources

We will search the following electronic databases since the inception date up to February 27, 2019: MEDLINE (PubMed), CENTRAL, and EMBASE (Elsevier, EMBASE.com) using the appropriate thesaurus and free-text terms. We will contact investigators and relevant trial authors, seeking information on unpublished data, if necessary.

We will check the reference lists of all the studies identified, and we will examine the references of any systematic review or meta-analysis identified during the search process.

No restriction on language or publication period will be applied. Non-English studies for which a translation cannot be obtained will be classed as potentially eligible but will not be considered in the full review. A full electronic search strategy for PubMed/MEDLINE is presented in Additional file 2.

### Study selection

Two of the authors of the present protocol will independently screen the abstracts of all the publications obtained by the search strategy. These authors will then independently assess the full text of the potentially relevant studies for inclusion. We will discard all studies that do not fulfill the above inclusion criteria. We will then obtain the full text of the remaining articles. We will resolve disagreements through discussion and consult a third author if disagreements persist. Covidence software [37] will be used to manage the study selection phase.

### Data extraction

We will use a specifically designed and piloted data collection form using an Excel sheet (Microsoft Inc.). Two authors will independently extract characteristics and outcome data from the included studies. Disagreements will be resolved through discussion or with assistance from a third author if necessary.

From each study included, we will extract the following variables expressed in PICO terms: Population definition (acute/subacute), number, gender and age of participants, dropouts; Interventions and Controls with details of treatment description (such as duration of whole treatment); and Outcomes (primary and secondary) with relative measurement scales and time point follow-up. Moreover, we will extract the following trial characteristics: name of the first author, year of publication, setting, number of centers, and funding sources.

All relevant arm-level data will be extracted. For pain and disability outcomes, we will consider post-treatment assessments. When these are lacking, the post-treatment data will be extrapolated by the difference between the baseline and mean change values and SDs will be imputed using the average of the available SD for the same instrument within the same network [38]. If there will be enough information, we will perform a secondary analysis using mean change and discuss possible differences. The AEs will be extracted as absolute number when available.

We will assume that any patient meeting the inclusion criteria is, in principle, equally likely to be randomized to any of the eligible low back pain interventions.

### Geometry and feasibility of the network

We will explicitly describe the process leading to node grouping [39, 40]. The network of treatments will be judged based on the characteristics of the available studies, presented and evaluated graphically. We will evaluate the following: if the network is disconnected; if there is a sufficient number of comparisons in the network with available direct data; if there is a high number of comparisons based on a single study; and if any key treatment is missing. Next, the feasibility of the network meta-analysis will be assessed checking the following: (i) transitivity (i.e., comparable distribution of effect modifiers across comparisons), which will be examined using boxplots or percentages to visually inspect potential effect modifiers of treatment effect [41]; (ii) consistency between direct and indirect estimates of the effects, which will be examined using the node-splitting method [42], and globally (i.e., evaluating the network as a whole), using the design-by-treatment interaction model [43]; and (iii) the amount of variability, which we will quantify, that can be attributed to heterogeneity and inconsistency rather than sampling error, by calculating the *I*^2^ statistic [44].

All RCTs reporting only two arm comparisons between the same kind of intervention (e.g., exercise versus exercise) will be excluded, whereas if they present at least one third arm comparator, they will be included (e.g., exercise versus NSAIDs). We will include both multi-arm trials comparing three or more interventions and those comparing different dosages or regimens of an intervention to a different one. Intervention arms of different dosages and regimens of the same intervention will be merged together for the global analysis of all outcomes. We will not consider all the comparisons in which an intervention presents multiple co-interventions for the experimental group (e.g., mixed treatment: laser therapy plus manipulation plus exercise versus waiting list controls) or for the control group (e.g., usual care: education, some physical exercise plus drugs taken as needed) to avoid inconsistencies across trials.

### Risk of bias within individual studies

Two review authors will independently assess the risk of bias in the included studies. Disagreements will be resolved through discussion or arbitration with a third review author when consensus cannot be reached. We will assess the risk of bias for each included study using the “risk of bias” (RoB) assessment tool recommended by The Cochrane Collaboration [28]. Specifically, we will evaluate the following criteria: random sequence generation, allocation concealment, blinding of participants, providers and outcome assessment, incomplete outcome data, and selective outcome reporting. Each item will be scored as “high,” “low,” or “unclear” RoB if no sufficient information is reported. To summarize the overall RoB for a study, allocation concealment, blinding of outcome assessment, and incomplete outcome data will be carefully considered in order to classify each study as “low risk of bias” when all three criteria are met, “high risk of bias” when at least one criterion is unmet, and “moderate risk of bias” in the remaining cases. Allocation concealment, blinding of outcome assessment, and incomplete outcome data are not expected to vary in importance across the primary outcomes, and therefore, we will summarize the RoB of each study. RoB information will be used to interpret how risk of bias can affect data per each comparison in the network plot and in the interpretation of the quality of evidence.

### Quality of evidence

We will assess the certainty of evidence contributing to network estimate of the main outcomes with the Grading of Recommendation Assessment, Development and Evaluation (GRADE) framework. We considered the five GRADE domains: study limitations for RoB assessment, indirectness, inconsistency, imprecision, and publication bias [45].

### Measures of treatment effect

#### Methods for direct treatment comparisons

We will perform conventional pairwise meta-analyses for each primary outcome using a random-effects model for each treatment comparison with at least two studies [46] using Stata software v. 15 and the command metan [47].

We will estimate the primary outcomes as continuous outcomes, using the mean difference (MD) or standardized mean difference (SMD) when different outcome measurements have been reported for each trial. The uncertainty of all estimates will be expressed with its 95% confidence interval (CI).

#### Methods for multiple comparisons

We will estimate the primary outcomes as continuous outcomes, using standardized mean difference (SMD) as we expect multiple scales to be used. We will perform the network meta-analyses within a frequentist setting, assuming equal heterogeneity across all treatment comparisons and accounting for correlations induced by multi-arm studies [41, 48]. We will use a multivariate normal model with random effects [43]. We will first fit a design by treatment interaction model to assess the presence of inconsistency (global *χ*^2^ test). If the null hypothesis of all inconsistency parameters being equal zero is not rejected, we will fit a consistency model. If a global significant inconsistency is found, we will try to interpret the significant inconsistency parameters, split nodes to possibly remove the problem, and try to model the inconsistency using meta-regression. If there will be enough information, we will perform a secondary analysis using mean change and discuss possible differences.

#### Relative treatment ranking

We will estimate all ranking probabilities and cumulative ranking probabilities for each treatment and outcome. We will then calculate the median rank with their 95% credible intervals, to assess the robustness of the finding. To determine a treatment hierarchy with a single number, we will calculate the surface under the cumulative ranking curve (SUCRA) and express it as a percentage [49]. Presenting the results with this method will help to visualize the relative efficacy of treatments, as it will provide the probabilities for a treatment to be *i*th ranking (i.e., first, second, third), for each possible rank, in improving the outcome of interest. We will perform network meta-analyses in Stata 15 [47] using the “network” command and the “mvmeta” command [43, 50–52].

### Assessment of statistical heterogeneity

In the standard pairwise comparisons, we will assess the statistical heterogeneity within each pairwise comparison using the *I*^2^ statistic, where an *I*^2^ value of 25 to 49% indicates a low degree of heterogeneity, 50 to 75% a moderate degree of heterogeneity, and more than 75% indicates a high degree of heterogeneity [53].

In the network meta-analyses, we will assume that the standard heterogeneity is constant across the different treatment comparisons. We will estimate it including a random effect in the multivariate normal model, assuming a multivariate normal distribution with mean 0 and a variance-covariance matrix with diagonal elements *τ*^2^ and off-diagonal elements equal to *τ*^2^/2 and discuss the magnitude of the estimated variance parameter.

### Assessment of transitivity and statistical consistency in network meta-analyses

We will assess the assumption of transitivity (or similarity) by comparing the distribution of the potential effect modifiers across the various pairwise comparisons. If there are no multi-arm trials, we will evaluate the inconsistency assumption in each closed loop of the network separately as the difference between direct and indirect estimates for a specific comparison (inconsistency factor). The magnitude of the inconsistency factors and their 95% CIs will be used to make an inference about the presence of inconsistency in each loop.

If multi-arm trials are present, as it is problematical to identify loop inconsistencies, we will use the node-splitting approach to evaluate existing differences between direct and indirect estimates for each node [42].

To check the assumption of consistency in the entire network, we will use the design-by-treatment model as described by Higgins [43]. This method accounts both for loop and design (i.e., different sets of treatments compared in a trial) inconsistencies in multi-arm trials. Using this approach, we will make an inference about the presence of inconsistency from any source in the entire network based on an *χ*^2^ test. Inconsistency and heterogeneity are interwoven: to distinguish between these two sources of variability, we will employ the *I*^2^ statistic for inconsistency, as it measures the percentage of variability that cannot be attributed to random error or heterogeneity (within comparison variability).

If heterogeneity is identified and at least 10 studies are present, we will conduct a meta-regression analysis to explain the observed heterogeneity [54]. The meta-regression analysis will explore the following factors as the most likely sources of inconsistency between direct and indirect evidence: baseline pain values (source of statistical heterogeneity); age, gender, patients with acute and subacute pain (sources of clinical heterogeneity); and study quality (source of methodological heterogeneity).

### Sensitivity analysis

We will provide sensitive analysis in the situation when (i) outlying studies are present and suspected and (ii) studies are arbitrarily grouped. Moreover, in case of more than 10 studies available, we will assess a small study effect for each outcome providing an adjusted funnel plot and using *netfunnel* command (Stata 15.0 software).

## Discussion

Our systematic review results will have a direct impact on a large proportion of the population affected by non-specific LBP since this is the most leading cause of disability worldwide. The comparative efficacy among different therapeutic interventions for acute non-specific LBP, including pharmacological and physiotherapy treatments, is currently unknown. Indeed, the results will influence therapeutic strategies for patients with LBP, policymakers and all stakeholders.

Our review has several strengths including (I) exploring a wider range of literature databases including eligible articles in all languages, (II) a transparent reporting of description of interventions for a consistent node decision making, and (III) the plan to present summary assessments using the GRADE approach to rate the quality of evidence ensuring transparent reporting and clearer interpretation of results.

We anticipate that our included interventions can be a proxy of the actual clinical practice since we will be highly selective excluding all combined interventions. Indeed, the inclusions of mixed interventions (i.e., ultrasound plus exercise) can be uninformative of which is the effective part of the treatment. This is a common problem of complex non-pharmacological interventions [55, 56]. Potential issues of the proposed review include high clinical heterogeneity, poor quality of reporting of the included trials, and difficulty in interpreting measures of effect when the pooled estimates come from trials that measured the outcome using different measurement tools [35, 57]. Another plausible limitation, solely concerning the network meta-analysis, might be the lack of available treatment comparisons to build robust nodes.

Any important protocol amendments will be transparently documented. We aim to disseminate the results of the NMA: we will publish the findings in an open access journal, present them at scientific conferences, and conduct dissemination meetings with key stakeholders (including policymakers and healthcare providers). We will also consider dissemination through social media tools.

## Additional files

Additional file 1: PRISMA-P checklist. (DOCX 30 kb)

Additional file 2: PubMed search strategy and adapted for the other databases. (DOCX 14 kb)

## Abbreviations

DALYs: Disability-adjusted life-year
LBP: Low back pain
MD: Mean difference
NMA: Network meta-analysis
NSAIDs: Non-steroidal anti-inflammatory drugs
PRISMA-P: Preferred Reporting Items for Systematic Reviews and Meta-Analyses Protocol
RCT: Randomized controlled trial
RoB: Risk of bias
SD: Standard deviation
SMD: Standardized mean difference
SUCRA: Surface under cumulative ranking area
