# Supplementary material for: Effectiveness of treatments for acute and sub-acute mechanical non-specific low back pain: protocol for a systematic review and network meta-analysis
Source: Syst Rev. 2019 Aug 8;8:196. doi: 10.1186/s13643-019-1116-3 (PMC6688358; doi:10.1186/s13643-019-1116-3)
Supplement: Supplementary file 2 — PubMed search strategy and adapted for the other databases. (DOCX 14 kb) [file 13643_2019_1116_MOESM2_ESM.docx]

**Additional file 1:** PubMed search strategy

1. "Adult"[Mesh] OR adult*[Title/Abstract]

2. back pain[Mesh] OR “low back pain” [Title/Abstract] OR “back pain”[Title/Abstract] OR backache [Title/Abstract] OR lumbago [Title/Abstract] OR “back disorder”[Title/Abstract]

3. Randomized Controlled Trial[ptyp] OR Controlled Clinical Trial[ptyp] OR RCT [Title/Abstract] OR “Randomized Controlled Trial” [Title/Abstract] OR random* [Title/Abstract] OR trial[Title/Abstract]

4. “Exercise”[Mesh] OR “Exercise therapy”[Mesh] OR Exercis* [Title/Abstract] OR training[Title/Abstract] OR “motor control” [Title/Abstract] OR “back school” [Title/Abstract] OR Manipulation, Chiropractic [Mesh] OR Manipulation, Orthopedic[Mesh] OR Manipulation, Osteopathic [Mesh] OR Manipulation, Spinal [Mesh] OR Musculoskeletal Manipulations [Mesh] OR Chiropractic [Title/Abstract] OR manipulation [Title/Abstract] OR manipulate[Title/Abstract] OR “Spinal Manipulation” [Title/Abstract] OR “Lumbar Manipulation” [Title/Abstract] OR thrust [Title/Abstract] OR manual therap* [Title/Abstract] OR mobilization [Title/Abstract] OR Acupuncture [Mesh] OR Acupuncture Therapy [Mesh] OR acupuncture [Title/Abstract] OR electro-acupuncture [Title/Abstract] OR acupressure [Title/Abstract] OR dry-needling [Title/Abstract] OR Massage[Mesh] OR massage [Title/Abstract] OR “myofascial release” [Title/Abstract] OR Trigger Points [Mesh] OR “Trigger Points” [Title/Abstract] OR Health Education

[Mesh] OR “Physical Education and Training”[Mesh] OR patient education [Mesh] OR "Patient-Centered Care"[Mesh] OR “information booklet” [Title/Abstract] OR book* [Title/Abstract] OR pamphlet* [Title/Abstract] OR leaflet* [Title/Abstract] OR poster* [Title/Abstract] OR education* [Title/Abstract] OR information* [Title/Abstract] OR Diathermy[Mesh] OR Diatherm* [Title/Abstract] OR tecar[Title/Abstract] OR Transcutaneous Electrical Nerve Stimulation [Mesh] OR TENS [Title/Abstract] OR “Electric Nerve Stimulation” [Title/Abstract] OR “Electrical Stimulation therapy” [Title/Abstract] OR Electrostimulation [Title/Abstract] OR “electric stimulation therapy” [Title/Abstract] OR electroanalgesia [Title/Abstract] OR electroacupuncture [Title/Abstract] OR electromagnetic [Title/Abstract] OR electrotherapy*[Title/Abstract] OR taping [Title/Abstract] OR tape*[Title/Abstract] OR Kinesio[Title/Abstract] OR strap*[Title/Abstract] OR sound [Mesh] OR Ultrasonic Therapy [Mesh] OR

Ultrasonics [Mesh] OR Ultrasonography [Mesh] OR Ultrasonic Waves [Mesh] OR Ultrasonic* [Title/Abstract] OR ultrasound [Title/Abstract] OR Low-Level Light Therapy [Mesh] OR laser [Title/Abstract] OR infrared [Title/Abstract] OR ultraviolet [Title/Abstract] OR monochromatic [Title/Abstract] OR drug therapy [Title/Abstract] OR NSAIDS[Title/Abstract] OR "Cyclooxygenase Inhibitors" [Pharmacological Action] OR cyclooxygenase [Title/Abstract] OR cyclo-oxygenase [Title/Abstract] OR Anti-Inflammatory Agents, Non-Steroidal [Pharmacological Action] OR Anti-Inflammatory Agents, Non-Steroidal [Mesh] OR aspirin [Title/Abstract] OR acetylsalicyl* [Title/Abstract] OR Salicylic Acid [Title/Abstract] OR carbasalate calcium [Title/Abstract] OR Diflunisal[Title/Abstract] OR aceclofenac [Title/Abstract] OR alclofenac [Title/Abstract] OR Diclofenac [Title/Abstract] OR Indomethacin [Title/Abstract] OR Sulindac [Title/Abstract] OR meloxicam [Title/Abstract] OR Piroxicam [Title/Abstract] OR dexibuprofen [Title/Abstract] OR dexketoprofen [Title/Abstract] OR Fenoprofen [Title/Abstract] OR Flurbiprofen [Title/Abstract] OR ibuprofen [Title/Abstract] OR ketoprofen [Title/Abstract] OR Naproxen[Title/Abstract] OR metamizol [Title/Abstract] OR Dipyrone[Title/Abstract] OR phenylbutazone [Title/Abstract] OR phenazone [Title/Abstract] OR Antipyrine[Title/Abstract] OR propyphenazone [Title/Abstract] OR celecoxib[Title/Abstract] OR etoricoxib [Title/Abstract] OR nabumeton[Title/Abstract] OR parecoxib[Title/Abstract] OR rofecoxib [Title/Abstract] OR celecoxib [Title/Abstract] OR valdecoxib [Title/Abstract] OR lumiracoxib[Title/Abstract] OR etoricoxib [Title/Abstract] OR parecoxib [Title/Abstract] OR vioxx[Title/Abstract] OR celebrex[Title/Abstract] OR bextra[Title/Abstract] OR prexige[Title/Abstract] OR arcoxia [Title/Abstract] OR etodolac [Title/Abstract] OR floctafenine[Title/Abstract] OR Meclofenamic Acid[Title/Abstract] OR meclofenamate [Title/Abstract] OR

meloxicam[Title/Abstract] OR oxaprozin[Title/Abstract] OR piroxicam[Title/Abstract] OR tenoxicam[Title/Abstract] OR tolmetin[Title/Abstract] OR paracetamol[Title/Abstract] OR Acetaminophen[Mesh] OR “Analgesics, Opioid”[Pharmacological Action] OR M03$ [Title/Abstract] OR muscle relax*[Title/Abstract] OR anti-spasm* [Title/Abstract] OR calmative[Title/Abstract] OR carisoprodol[Title/Abstract] OR cyclobenzaprine[Title/Abstract] OR flexeril[Title/Abstract] OR metaxalone[Title/Abstract] OR methocarbamol[Title/Abstract] OR baclofen[Title/Abstract] OR orphenadrine[Title/Abstract] OR tizanidine[Title/Abstract] OR Zanaflex[Title/Abstract] OR dantrolene[Title/Abstract] OR Dantrium[Title/Abstract] OR Quinine[Title/Abstract] OR chlorzoxazone[Title/Abstract] OR norflex[Title/Abstract] OR norgesic[Title/Abstract] OR alprazolam[Title/Abstract] OR xanax[Title/Abstract] OR Triazolam[Title/Abstract] OR Brotizolam[Title/Abstract] OR Oxazepam[Title/Abstract] OR

Loprazolam[Title/Abstract] OR Lormetazepam[Title/Abstract] OR Lorazepam[Title/Abstract] OR Ativan[Title/Abstract] OR Temazepam[Title/Abstract] OR Normison[Title/Abstract] OR Temaz*[Title/Abstract] OR Estazolam[Title/Abstract] OR Bromazepam[Title/Abstract] OR Chlordiazepoxide[Title/Abstract] OR Clobazam[Title/Abstract] OR Nimetazepam[Title/Abstract] OR Flunitrazepam[Title/Abstract] OR Nitrazepam[Title/Abstract] OR Clonazepam[Title/Abstract] OR Quazepam[Title/Abstract] OR Diazepam[Title/Abstract] OR Valium[Title/Abstract] OR Phenazepam[Title/Abstract] OR Medazepam[Title/Abstract] OR Prazepam [Title/Abstract] OR Flurazepam[Title/Abstract] OR Clorazepate[Title/Abstract] OR Nordazepam[Title/Abstract] OR NO2A*[Title/Abstract] OR opioid [Title/Abstract] OR analges*[Title/Abstract] OR narcotic*[Title/Abstract] OR morphine[Title/Abstract] OR ordine [Title/Abstract] OR hydromorphone [Title/Abstract] OR dilaudid[Title/Abstract] OR

oxycodone[Title/Abstract] OR endone[Title/Abstract] OR targin[Title/Abstract] OR oxymorphone[Title/Abstract] OR OPANA*[Title/Abstract] OR codeine[Title/Abstract] OR dihydrocodeine[Title/Abstract] OR ketobemidone[Title/Abstract] OR pethidine[Title/Abstract] OR Fentanyl[Title/Abstract] OR durogesic[Title/Abstract] OR diphenylpropylamine[Title/Abstract] OR dextromoramide[Title/Abstract] OR piritramide[Title/Abstract] OR dextropropoxyphene[Title/Abstract] OR bezitramide[Title/Abstract] OR methadone[Title/Abstract] OR physeptone[Title/Abstract] OR pentazocine[Title/Abstract] OR phenazocine[Title/Abstract] OR buprenorphine[Title/Abstract] OR norspan[Title/Abstract] OR suboxone[Title/Abstract] OR subutex[Title/Abstract] OR etorphine[Title/Abstract] OR tilidine[Title/Abstract] OR trama*[Title/Abstract] OR tramadol[Title/Abstract] OR dezocine[Title/Abstract] OR tapentadol[Title/Abstract] OR meptazinol[Title/Abstract] OR

“benzodiazepines”[Mesh] OR injection* OR cortisone[Title/Abstract] OR prednisone[Title/Abstract] OR prednisolone[Title/Abstract] OR oral glucocorticosteroid [Title/Abstract] OR glucocortisone [Title/Abstract] OR steroid*[Title/Abstract] OR glucocorticoid* [Title/Abstract] OR corticosteroid*[Title/Abstract] OR glucosteroid*[Title/Abstract] OR cyclocosteroid*[Title/Abstract] OR cortiso* OR "Steroids"[Mesh] OR Betamethasone [Title/Abstract] OR Dexamethasone[Title/Abstract] OR Hydrocortisone[Title/Abstract] OR Methylprednisolone[Title/Abstract] OR Deflazacort[Title/Abstract]

5. 1 AND 2 AND 3 AND 4
